# Characteristics, Possible Origins, and Health Risk Assessment of Trace Elements in Surface Waters of the Han River Watershed, South Korea

**DOI:** 10.3390/ijerph192315822

**Published:** 2022-11-28

**Authors:** Jong Kwon Im, Yong Chul Cho, Young Seuk Kim, Soyoung Lee, Taegu Kang, Sang Hun Kim

**Affiliations:** Han River Environment Research Center, National Institute of Environmental Research, 42, Dumulmeori-gil 68beon-gil, Yangseo-myeon, Yangpyeong-gun, Incheon 12585, Gyeonggi-do, Republic of Korea

**Keywords:** water quality, trace element, Han River, health risk, hazard index, cancer risk

## Abstract

To safeguard aquatic environments in and around the Han River watershed in South Korea, a multivariate statistical evaluation of trace elements, a trace element concentration analysis and source determination, and a human health risk assessment were conducted on 10 trace elements at 25 sites. The results demonstrated that the Han River watershed was mainly affected by anthropogenic activities (traffic/industrial activity). The range of concentrations was arranged in descending order: Fe (217.13 ± 301.03 µg/L) > Mn (102.36 ± 153.04 µg/L) > Zn (23.33 ± 79.63 µg/L) > Ba (29.05 ± 12.37 µg/L) > Ni (5.14 ± 11.57 µg/L) > Cu (3.80 ± 3.56 µg/L) > Pb (0.46 ± 0.52 µg/L) > Se (0.06 ± 0.04 µg/L) > Cd (0.01 ± 0.01 µg/L) > Ag (0.004 ± 0.013 µg/L). The hazard index values of trace elements in surface water for combined pathways (ingestion and dermal contact) were < 1.0 for both adults and children, indicating no possible human health hazards. The estimated total cancer risk did not exceed the acceptable limit (1 × 10^−4^) for adults and children. The findings of this study provide data-driven guidelines for water environment policy decisions in the study area.

## 1. Introduction

Water is the most important natural resource for life on Earth; however, it is also the most misused [1]. As the water quality deteriorates, its usage by humans and other living organisms becomes challenging [2]; furthermore, any alteration in the biological, chemical, or physical quality of water adversely affects the health of human and other living organisms [3].

In urban areas, traffic and industrial activities are intensifying, owing to high population density and anthropogenic activity [4]. In such conditions, trace elements are emitted both indoors and outdoors by humans, mainly from coal combustion for heating and industry, transportation exhaust gas emissions, and raw sewage emissions [5]. Regional variability of anthropogenic activity induces spatial differences in trace element sources [6,7,8]. To safeguard water resources and reduce water pollution, it is crucial to elucidate the trace element distribution, concentration, and sources, while also estimating health risk levels and water quality [9,10].

Heavy metals (e.g., As, Cd, Cr, Cu, Mn, Ni, and Pb) are more likely to accumulate than degrade and can be transferred through the food chain [11]. Sources of heavy metal pollution can be divided into two pollution source categories: point pollution and non-point pollution [12]. Examples of point pollution sources include domestic sewage and effluent from factory wastewater treatment systems. Metals such as Cd, Cr, Cu, Fe, Mn, Ni, Pb, Sb, Ba, and Zn are commonly found in brake wear particles [13,14,15,16]. Similarly, tire wear debris can contain high concentrations of Cd, Co, Cr, Cu, Fe, Mn, Ni, Pb, and Zn [13]. Various research results such as Fe, Mn, Pb, Ni, and Zn have been published as rural nonpoint pollution sources [17,18,19]. 

Environmental studies can greatly benefit from using multivariate statistical techniques, such as factor/principal component analysis (FA/PCA) and correlation analysis. Unveiling hidden relationships between variables and reducing massive and complex chemical data sets into a small number of factors can help deepen our understanding of water quality and potential sources that can affect research systems [20,21,22].

However, as not all the trace element sources can be handled at once due to time and resource constraints, progressive management of the sources is more practical [23]. Consequently, identification of previously regulated sources can lay the foundation toward more effective pollution management and prevention [24]. Trace element contamination in aquatic ecosystems has attracted considerable attention from researchers because it can cause permanent harm to human health [20,25]. Trace elements are known to be systemic toxins, with teratogenic and carcinogenic effects, while also affecting many organs [26]. Additionally, the bioaccumulation of metals in dietary sources exposes people to trace elements via water sources [27]. 

Humans are frequently exposed to high quantities of trace elements from plant and aquatic food sources produced in polluted waters, even without directly ingesting trace element-contaminated water [28,29]. Trace elements may enter human bodies through the food chain, thereby accumulating in the body and posing a risk to health, either directly or indirectly [30]. Furthermore, humans may be exposed to trace metals via three major routes: oral and nasal inhalation, dermal absorption through skin contact, and direct ingestion. Water pathways frequently involve dermal absorption and drinking water consumption [31]. The degree of trace-element pollution in water is strongly associated with the health risks posed by chemical carcinogens [32]. Moreover, long-term exposure to trace elements even at low doses can be dangerous to humans [30]. To safeguard aquatic environments in and around the Han River watershed, this study aimed to investigate the level of trace element pollution in its water environment for potential health threats.

Thus, we collected 25 water samples from the Han River watershed (South Korea) and analyzed them for 10 trace elements. We aimed to: (1) determine the distribution of trace elements in surface water from the Han River watershed; (2) identify potential pollution sources and contribution ratios using principal component analysis (PCA) and correlation analysis; and (3) assess the carcinogenic and non-carcinogenic risks of trace elements through multiple exposure routes to surface water. The findings of this study can lay the theoretical foundation for water environment management and pollution control in the Han River watershed.

## 2. Materials and Methods

### 2.1. Study Area

**Figure 1:**
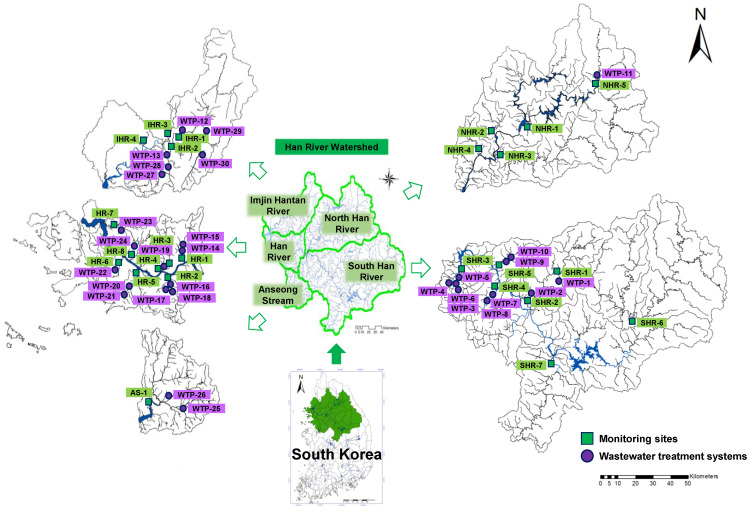
Han River watershed showing sampling and wastewater treatment system (WTSs) sites in five areas (HR, IHR, SHR, NHR, AS).

The Han River watershed is the largest watershed in Korea, which supplies drinking water to 26 million people. The area and length of the Han River watershed is 26,219 km^2^ and 5417 km, respectively. The Namhan and Bukhan rivers meet and flow from the Han River into the West Sea of South Korea. The Han River basin comprises covered forests and residential, commercial, agricultural, and factory areas. The Han River watershed is a subtropical region with a monsoon climate—high temperatures and high humidity in summer. Total precipitation in 2016 was 935.9 mm (2009–2016 average: 1428.8 mm), with 54% occurring between June and August (504 mm). Temperatures ranged between 7.0–19.6 °C from March to May (spring), 23.6–28.0 °C from June to August (summer), and 6.8–23.1 °C from September to November (fall). To identify the source of the surface water pollution, the region was divided into five areas based on geographical characteristics. The locations of all the sampling sites are shown in Figure 1.

### 2.2. Sampling Collection and Analysis

In April 2016, 25 water samples from the Han River basin were collected. The Han River basin was divided into five groups—NHR, SHR, HR, AS, and IHR. Fundamentally, the water quality samples were collected from upstream, middle, and downstream areas, and industrial, urban, and rural areas to investigate the effects of pollutants in each group.

After collection, the water samples were filtered using 0.45 um nylon filters and arranged for trace element analysis in a clean, high-density polyethylene container. The detailed pretreatment process of the samples is summarized in Figure S1.

Before the analysis, all the samples were stored at 4 °C. Then, the selected trace elements—Cd, Cu, Zn, Mn, Pb, Ni, Ag, Ba, Se, and Fe—were analyzed using inductively coupled plasma-mass spectrometry (ICP-MS, Agilent Technologies 7900 series, USA). The detection limits of all the target metals ranged from 0.002–0.233 μg/L. The method validation and quality control were performed using the standard reference materials (#5183-4688, Agilent, Santa Clara, CA, USA) [33]. All the samples, including the standard reference materials, were examined in batches; each contained a procedural blank. The recovery rates varied between 87.8% (Cu) and 110% (Ag) (Table S1). The R^2^ values of all the target metals exceeded 0.999 (Table S2).

### 2.3. Statistical Analysis Methods

The covariance matrix of the original variables was used to extract eigenvalues and eigenvectors using the factor analysis technique. Principal components (PCs), which are uncorrelated variables, were created by multiplying the original correlated variables by an eigenvector, which is a set of coefficients. The PCs were the weighted linear combination of the original variables. A computer provided the most crucial factors that could characterize the complete data set and compress the data with minimal loss of original information. This was a potent pattern recognition technique that sought to account for variance by condensing a huge set of highly correlated variables into a more manageable set of independent variables [15,34]. Fundamentally, dimensionality reduction is the central concept of PAC/FA, which can reduce a large number of indicators into a small number of synthetic indications that largely represent the original data [35]. To further reduce the impact of less significant variables, PCs were rotated using the maximum variance method to create new components (VFs). By computing the covariance, PCA can reveal the degree of dispersion of water quality parameters, obtain eigenvalues and eigenvectors, and retrieve principal components (PCs) [36]. 

The Bartlett spherical test was applied to determine whether the variable correlation coefficient matrix was an identity matrix. Furthermore, the KMO (Kaiser-Meyer-Olkin) and Bartlett tests were used to evaluate the appropriateness of the dataset for PCA/FA [37]. SPSS 22.0 (the Windows version) was used to perform the PCA/FA analysis. In this study, the following criteria were used: (2) the eigenvalues of VFs > 1 were selected; (1) the associated factor loadings at > 0.45 were highlighted [38].

### 2.4. Health Risk Assessment

The health risk assessment is an efficient, widely applied method for estimating the behavior and possibility of adverse health effects on humans as a result of exposure to trace elements through drinking water (ingestion) or the skin (dermal absorption) [39,40]. In this study, we used the US EPA’s (2004) [41] recommended health risk evaluation approach according to Equations (1) and (2). We computed the average daily dose (ADD) from oral consumption (ADD_oral_) and dermal absorption (ADD_dermal_).

(1) $${\mathrm{ADD}}_{\mathrm{oral}}=\frac{c_{w}\times \mathrm{IR}\times {\mathrm{ABS}}_{g}\times \mathrm{EF}\times \mathrm{ED}}{\mathrm{BW}\times \mathrm{AT}}$$

(2) $${\mathrm{ADD}}_{\mathrm{dermal}}=\frac{c_{w}\times \mathrm{SA}\times K_{p}\times \mathrm{ET}\times \mathrm{EF}\times \mathrm{ED}}{\mathrm{BW}\times \mathrm{AT}}$$

The oral absorption rate (2.0 and 0.64 L/day for adults and children, respectively) was represented as IR when the mean concentration of trace metals in a water sample was reported as C_w_. Fundamentally, ED reflects the exposure duration (70 and 6 years for adults and children, respectively). The terms BW and AT were utilized to describe the mean body weight (65 and 20 kg for adults and children, respectively) and the mean exposure duration (25, 550 and 2, 190 days for adults and children, respectively). In general, SA is the surface area (18,000 and 6600 cm^2^ for adults and children, respectively) of the exposed skin, ET is the exposure time (0.58 and 1.0 h/day for adults and children, respectively), ABS_g_ is the dimensionless gastrointestinal absorption factor, K_p_ is the unit of measurement of a water sample’s skin permeability coefficient (cm/h), and RfD is the oral reference dose (μg/kg/day) [41,42]. Table S3 summarizes the ABS_g_, K_p_, and RfD values.

The noncarcinogenic risk associated with oral trace element consumption was also estimated. To this end, the danger quotient was used for calculating the non-carcinogenic risk (HQ). The hazard index (HI) is the sum of the HQs, which quantifies the exposure trace elements potentially posing non-carcinogenic risk. Equations (3) and (5) were used to calculate HQ and HI [41]:

(3) $$\mathrm{HQ}=\frac{\mathrm{ADD}}{\mathrm{RfD}}$$

(4) $${\mathrm{RfD}}_{\mathrm{dermal}}=\frac{{\mathrm{RfD}}_{\mathrm{oral}}}{{\mathrm{ABS}}_{g}}$$

(5) $$\mathrm{HI}=\left({\mathrm{HQ}}_{\mathrm{oral}}+{\mathrm{HQ}}_{\mathrm{dermal}}\right)$$

Negative effects on people’s health may occur if the HQ or HI value is greater than one; however, a value of less than one indicates no negative effects.

The risk of developing cancer throughout a lifetime as a result of exposure to or contact with trace elements is known as carcinogenic risk (CR). Equation (6) can be used to calculate the CR.

(6) $$\mathrm{CR}=\left(\mathrm{ADD}+\mathrm{SF}\right)$$

where SF implies the oral cancer slope factor (μg/kg/day)^−1^. In this study, the CR was evaluated for Cd, Pb, and Ni; and the slope factors were 0.0061, 0.0085, and 0.00084 (μg/kg/day)^−1^ for ingestion intake, respectively [43,44].

## 3. Results and Discussion

### 3.1. Distribution of Trace Elements along the River

The overall and spatial distributions of Ag, Ba, Cd, Cu, Fe, Mn, Ni, Pb, Se, and Zn in the surface waters of 25 sites in the Han River watershed are presented in Table 1. In general, concentrations of Ag, Ba, Cd, Cu, Fe, Mn, Ni, Pb, Se, and Zn ranged from 0.002–0.036, 8.150–65.430, 0.003–0.035, 0.290–16.210, 30.430–1453.09, 8.670–729.970, 0.380–57.730, 0.050–2.510, 0.060–0.190, and 0.500–404.420 μg/L, respectively. 

**Figure 2:**
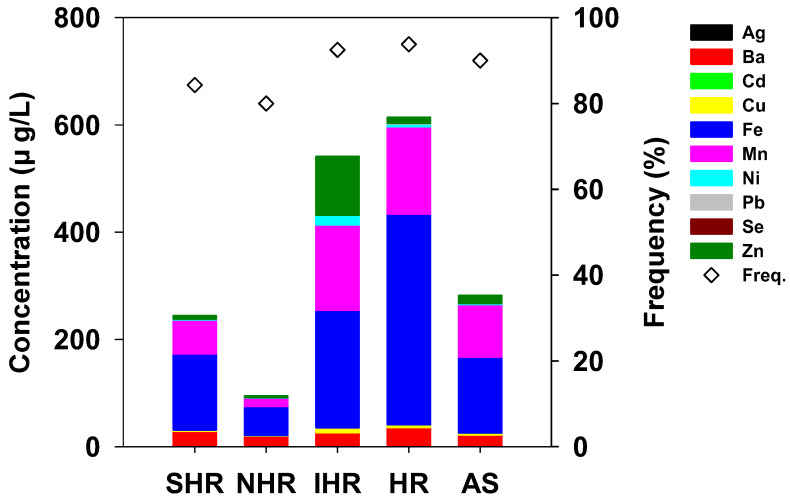
Cumulative concentration and detection frequency at five areas in the Han River watershed.

The spatial distribution characteristics of the 10 elements along the surface water revealed 10 trace elements in five groups of areas, which exhibited different patterns of distribution, with significant variations in the 25 sampling sites, thus indicating that these elements had different sources. The order of the total concentrations of each trace element in the five areas was revealed as follows: HR > IHR > AS > SHR > NHR (Figure 2). Moreover, Mn, Pb, Ba, Se, and Fe exhibited higher concentrations in the HR area, while Cu, Zn, Ni, and Ag were identified in the IHR area, and higher concentrations of Cd were found in the AS area. The HR area represents the capital of South Korea (Seoul); a megacity with the population of ~10 million, a highly economically developed area. In Seoul, industrial complexes are concentrated in the IHR area, while AS is a rural area with factories and wastewater treatment systems (WTSs) located near the stream. The relatively high concentrations of trace elements detected in these areas can be attributed to this spatial pattern. Overall, the varied trace element distribution features were driven by the geographical heterogeneity of anthropogenic activities (industrial, agricultural, and domestic) and economic growth in various areas of the Han River watershed.

The water pollution was further evaluated by comparing trace element concentrations against the standards for drinking water set by the WHO and the U.S. EPA, and the EU, India, Canada, Japan, China, and South Korea (Table 2). We found that the concentrations of Cd, Cu, Zn, Pb, Ag, Ba, and Se all met drinking water standards. However, the average concentration of Mn (102.4 μg/L) exceeded all the other drinking water standards. It ranged from 50–100 μg/L, except for by Canada’s standard (120 μg/L). At the HR-8 site, the peak concentration of Mn was found to be 730 μg/L, exceeding 7.3 times of the WHO, India, and China standards, 14.6 times that of the U.S. EPA, the EU, Japan, and South Korea standards, and 6.1 times that of Canada. At all 25 sites, the minimum concentration of drinking water standards exceeding 50 μg/L was 56% at 14 sites; these estimates were applicable to most HR, IHR, and SHR areas. However, the concentrations at the sites in the NHR area were all below the drinking water standard, ranging from 8.67–24.51 μg/L. 

Overall, the average concentration of Fe (217.13 μ/L) was less than 300 μg/L, which is the drinking water standard, except for the EU drinking water standard of 200 μg/L. The highest concentration of Fe was 1453.1 μg/L at HR-8, thereby manifesting the same conditions as the highest concentration of Mn. The latter exceeded the WHO, the U.S. EPA, India, Canada, Japan, China, and South Korea standards by 4.8 times, and 7.3 times that of the EU. Indeed, nine out of the twenty-five sites exceeded (36%), but no excess concentration was detected in the NHR area.

The average Ni was 5.14 μg/L, thereby manifesting values below the drinking water standards of each country, but the highest concentration of 57.7 μg/L exceeded all other standards (10–20 μg/L), except the WHO standard. Of all the sites, only two, IHR-1 and HR-1, exceeded the Ni drinking water standard. However, the drinking water standards in South Korea, the U.S. EPA, and Canada for Ni have not yet been established. The results of the heavy metal concentrations observed in this study were similar to [45], or slightly lower than [46,47] the concentrations found in studies that were based in other countries.

The majority of the enriched sites with trace metals were identified in the HR and IHR areas. This pattern was most likely driven by the substantial anthropogenic activity along the river. Given the predominant toxic pollutants in water systems, even their low concentrations should be strictly controlled and monitored.

### 3.2. Correlation Matrix

Fundamentally, a correlation matrix reflects the overall coherence of the dataset, thereby elucidating the relationships between variables [48,49]. The relationships between the ten trace elements were determined here using a correlation matrix. Strong positive correlations (*p* < 0.01) were discerned between each pair of trace elements, as shown in Table 3 [50]. The correlation values ranged from 0.528–0.846, while Cu and Zn, Cu and Ni, Mn and Ni, Mn and Fe, and Pb and Fe exhibited extremely strong significant correlations (R^2^ > 0.8), thus indicating comparable behavior or the same origins [51].

Co-occurrence sheds light on the pathways and sources of the components. Moreover, the indication of co-occurrence can be applied to reduce the number of target metals, thereby offering better economic conditions from time and cost standpoints.

### 3.3. Factor and Principal Component Analysis

We narrowed the dataset down to a number of influencing factors, based on which, PCA was conducted to investigate the sources of the trace elements [10]. We also used FA with a varimax rotation approach, the factor contribution of variables, while identifying the minor significance using PCA, which were further decreased. Kaiser-Meyer–Olkin (KMO) and Bartlett’s sphericity tests were applied to determine whether the data were appropriate for the factor analysis or not [17]. The significance of the KMO and Bartlett’s sphericity tests were found to be 0.001. The dataset was initially standardized using the z-scale transformation before applying FA/PCA to prevent the numerical ranges of original variables [48,49]. Table 4 illustrates the FA/PCA results, including eigenvalues, variance, and communalities.

**Figure 3:**
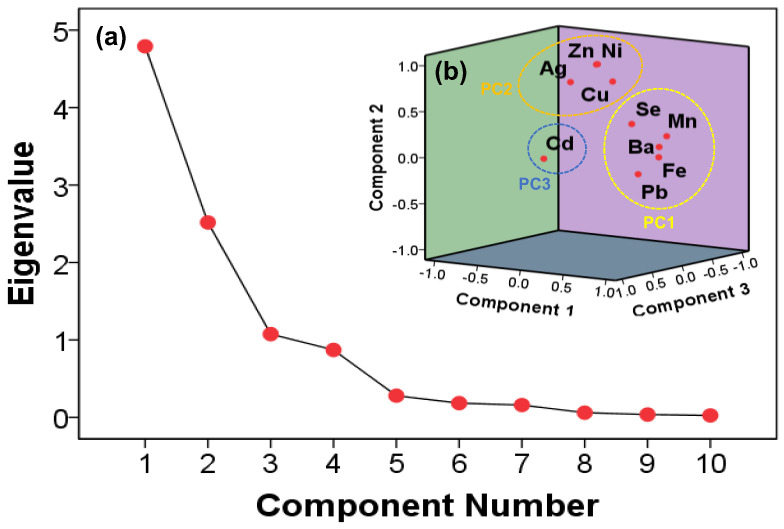
Principal component analysis (PCA) of 10 trace elements by (**a**) scree plot of the characteristic roots (eigenvalue) and (**b**) component plot in rotated space.

Three independent factors, each with an eigenvalue above 1, were extracted from the analysis, accounting for 83.82% of the overall variance (Figure 3). According the absolute loading values of 0.75 and above, 0.75–0.50, and 0.50–0.30, the factor loadings were categorized as “strong”, “moderate”, and “weak”, respectively [52,53]. Strong positive loadings for Pb (0.87), Ba (0.87), Mn (0.92), and Fe (0.95) were discerned in the first component, which explained 36.3% of the total variance with the weak positive loading for Se (0.42). A total of 36.295%, 35.055%, and 12.471% of the variance was attributed to each factor 1 to 3.

The elements of factor 1 were relatively high in the HR area, with high population density and economic center, and the IHR, with industrial complexes. The significant concentrations of Mn, Fe, Pb, and Ba can be attributed to the roadside soils in the urban areas [16]. Notably, Mn is mainly found in automobile fuel [54], while Fe is arguably a component of automobile brake pads [55]. Moreover, Pb and Ba have been previously reported to be highly correlated with asphalt wear [16]. In contrast, a low concentration of Pb was reported, despite Pb being banned as a fuel additive in South Korea in 1993 [56]. Hence, Pb can be assumed to be generated from road paint components instead [16,55]. Furthermore, Se is mainly generated from the wastewater treatment in manufacturing facilities and industrial processes [57,58].

Factor 2, which explained 35.06% of the total variance, exhibited positive correlation with Ni (0.97), Zn (0.96), Ag (0.84), and Cu (0.84). Notably, Ni, Zn, and Cu are metals whose sources are the wear of brakes, tires, and asphalt by vehicles in urban activities [59,60]. However, previous studies have reported non-point pollution source in soil as a component of fertilizer [18]. Ayrault et al. (2013) [61] confirmed that Ag emerged due to the erosion of the contaminated soil rather than due to discharge from the wastewater facility. Moreover, Birmili et al. (2006) [62] examined atmospheric PM in vehicles and concluded that Ag stemmed from a minor effect by vehicles. Therefore, Factor 2 can be deemed as a source of pollution from urban and agricultural activities.

An additional 12.47% of the total variance was explained by Factor 3, with Cd (0.93) contributing the most to this variance. However, relatively low concentrations of Cd were detected. Notably, previous studies indicated that it mainly occurred in urban and forest areas [17]. However, it can be assumed that these activities related to Cd are limited in the Han River watershed.

### 3.4. Risk Assessment on Human Health

**Figure 4:**
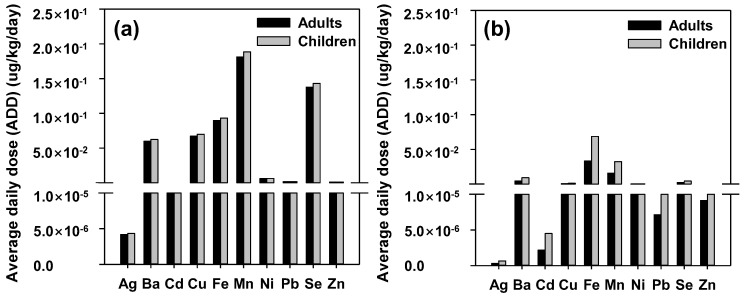
Average daily dose (ADD) for both (**a**) ingestion rate and (**b**) dermal adsorption of adults and children in the Han River watershed.

**Figure 5:**
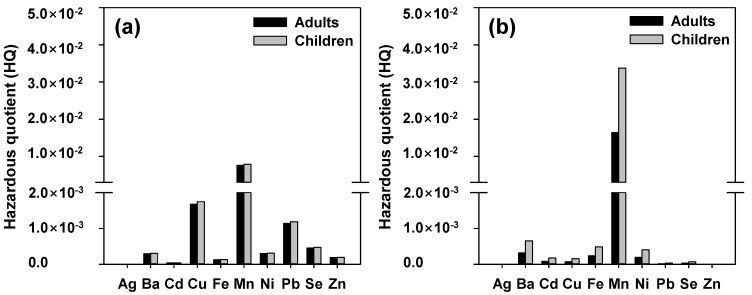
Hazardous quotient (HQ) for both (**a**) ingestion rate and (**b**) dermal adsorption of adults and children in the Han River watershed.

To quantify the negative impact of human exposure via the two separate methods of ingestion and dermal routes, the metal concentration in river water was further used. At this step, the ADD, HQ, and HI estimations were configured using the CR determination regarding the specific key paths. Figure 4 and Figure 5 show the average ADD with HQ computed values for adults and children, respectively. The ADD values for adults through the dermal and ingestion routes were arranged in the following descending order: Fe > Mn > Ba > Zn > Cu > Ni > Se > Pb > Cd > Ag and Mn > Zn > Fe > Cu > Ba > Ni > Se > Pb > Cd > Ag. Notably, these estimates are similar to previous findings from Alves et al. (2014) [63] and Mitra et al. (2018) [64], who revealed metal ingestion via the uptake pathway, thus indicating a dominant role for the ADD of all metals. The results also demonstrated that Mn was the most consumed and absorbed metal, averaging 1.81 × 10^−1^ μg/kg/day via ingestion, and that Fe was ingested at a rate of 3.34 × 10^−2^ μg/kg/day via dermal absorption. Moreover, children exhibited the highest consumption and absorption rates for Mn (1.88 × 10^−1^ μg/kg/day) via the ingestion route and Fe (6.87 × 10^−2^ μg/kg/day) via the dermal route.

HQ quantifies the non-carcinogenic risk through various important pathways for adults and children. The following rankings were identified for the mean HQ values across all age groups in Figure 5: Mn, Cu, Pb, Zn, Ni, Ba, Se, Fe, Cd, and Ag (ingested), and Mn, Ba, Fe, Ni, Cd, Cu, Zn, Pb, Se, and Ag (dermal). Moreover, Mn exhibited values of 7.55 × 10^−3^ and 7.85 × 10^−3^ for adults and children, respectively, and the highest values of HQ (uptake) were identified, while Mn had the highest HQ (dermal), with values of 1.64 × 10^−2^ for adults and 3.37 × 10^−2^ for children.

**Figure 6:**
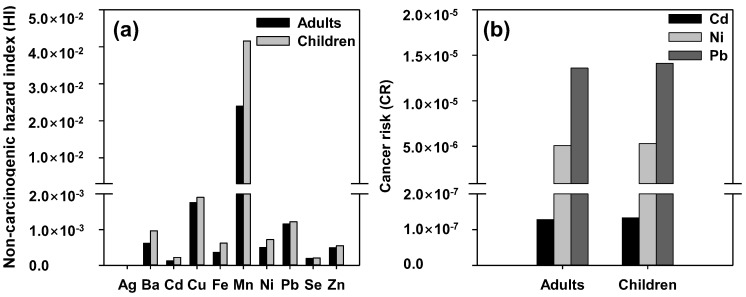
Non-carcinogenic hazard index (HI, (**a**)) and cancer risk (CR, (**b**)) of adults and children in the Han River watershed.

In a previous study, considering two different types of communities, the children’s HQ (ingestion) values were marginally higher than those of adults from Saha et al. (2017) [65]. Unexpectedly, unlike the ingestion and dermal contact, a more than two-fold difference between adults and children was identified. Namely, although the surface area (SA) of adults was three times larger, the average time (AT) was ~10 times larger, thus manifesting high values of the dermal HQ in children [66]. For both adults and children in the Han River watershed, the non-carcinogenic risk associated with combined ingestion and dermal exposure to Ag, Ba, Cd, Cu, Fe, Mn, Ni, Pb, Se, and Zn did not exceed the safety limit (HI < 1.0), as seen from Figure 6a. For both adults and children, Mn exhibited the highest HI, followed by Cu, Pb, Ba, Zn, Fe, Ni, Se, Cd, and Ag. As a result, the HI values below 1 for all the measured metals do not necessarily indicate a risk to human health. Notably, the HI value for children was 1.65 times higher than that for adults, thereby suggesting potentially higher vulnerability for children to non-carcinogenic risks from trace elements [67].

Figure 6b shows the CR calculation results for Cd, Ni, and Pb. As seen, the CR in children and adults from oral exposure to Cd, Ni, and Pb in the Han River basin did not exceed the safe range suggested by the USEPA (1.0 × 10^−6^ to 1.0 × 10^−4^). The CR values above 1 × 10^−4^ have been previously reported to pose a possible risk in humans [68]. However, Figure 6b shows that the risk of childhood cancer from water intake was below the limit for the investigated trace elements in the following order: Pb (1.41 × 10^−5^) > Ni (5.3 × 10^−6^) > Cd (1.33 × 10^−7^), with no evidence of any carcinogenic effects. The CR calculations for adults also revealed the same ranking for Pb (1.36 × 10^−5^) > Ni (5.09 × 10^−6^) > Cd (1.28 × 10^−7^), thus exhibiting a lower estimated cancer risk than children. Consequently, the studied trace elements were not significant enough to reduce cancer risk. Compared to the CR results of other studies [43,69,70], this study found a relatively high value of cancer-causing potential to be unlikely, while results obtained from another study [71] showed a similar value of non-carcinogenicity. 

## 4. Conclusions

The water quality and health risks of surface water from the five areas of the Han River watershed in South Korea were evaluated in terms of 10 trace element concentrations. The detection frequency range of the 10 trace elements was 24–100% and the mean concentration range was 0.004–217.128 μg/L in all of the analyzed samples. Although the concentrations of Cd, Cu, Zn, Pb, Ag, Ba, and Se met the drinking water standards, the highest concentrations of Fe and Ni detected at each site exceeded the EU and WHO drinking water standards, respectively, while the other standards were satisfied. None of the trace elements in the NHR area exceeded drinking water standards. Furthermore, the highest concentration of Mn exceeded all drinking water standards. The area with the highest concentration of total trace elements was HR, followed by IHR, AS, SHR, and NHR. PCA results identified three factors that accounted for 83.8% of the total variance, thus indicating that the Han River watershed was affected by anthropogenic sources, such as traffic and industrial activities. The HI values of all the trace elements did not exceed the safety limit (< 1), while the CR values of children and adults indicated the same ranking of Pb > Ni > Cd without evidence of carcinogenic effects. In conclusion, the concentrations of the examined trace elements in the Han River watershed exceeded some drinking water standards, but are likely insignificant to humans in terms of health risk. However, the low concentrations of trace elements should not be neglected. Our study provides data-driven guidelines for water environment policy decisions.

## Figures and Tables

**Table 1:** Statistical summary of target trace element concentration in the Han River watershed.

Compounds	Min Conc. (µg/L)	Max Conc. (µg/L)	Mean Conc. (µg/L)	Standard Deviation (µg/L)	Frequency Detected (%)
Ag	0.002	0.036	0.004	0.013	24
Ba	8.150	65.430	29.048	12.365	100
Cd	0.003	0.035	0.014	0.008	100
Cu	0.290	16.120	3.800	3.563	96
Fe	30.430	1453.090	217.128	301.030	100
Mn	8.670	729.970	102.364	153.044	100
Ni	0.380	57.730	5.139	11.567	100
Pb	0.050	2.510	0.463	0.524	100
Se	0.060	0.190	0.059	0.042	56
Zn	0.500	404.420	23.334	79.630	100

**Table 2:** Drinking water quality criteria in countries and by organizations (µg/L).

Compounds	South Korea	WHO	US EPA	EU	BIS	Canada	Japan	China
Ag			100 *		100			50
Ba		1300	2000		700	2000		700 *
Cd	5	3	5	5	3	7	3	5
Cu	1000 *	2000	1300	2000	1500 *	2000	1000	1000
Fe	300 *	300 *	300 *	200 *	300	300 *	300	300
Mn	50 *	100 *	50 *	50 *	100	120	50	100
Ni		70		20	20		10 *	20 *
Pb	10	10 *	15	10	10	5	10	10
Se	10	40 *	50	10	10	50	10	10
Zn	3000 *	4000 *	5000 *		5000	5000 *	1000	1000

* Based on aesthetic considerations.

**Table 3:** Correlation matrix of trace elements in the Han River watershed.

	Cd	Cu	Zn	Mn	Pb	Ni	Ag	Ba	Se
Cd	1								
Cu	0.293	1							
Zn	0.598 **	0.806 **	1						
Mn	0.29	0.788 **	0.653 **	1					
Pb	0.588 **	0.578 **	0.528 **	0.732 **	1				
Ni	0.385	0.846 **	0.771 **	0.812 **	0.542 **	1			
Ag	0.386	0.365	0.346	0.409*	0.537 **	0.362	1		
Ba	0.271	0.675 **	0.429 *	0.622 **	0.615 **	0.615 **	0.319	1	
Se	0.194	0.54 **	0.473	0.635 **	0.393	0.676 **	0.233	0.452 *	1
Fe	0.466 *	0.692 **	0.588 **	0.828 **	0.840 **	0.619 **	0.568 **	0.625 **	0.373

* 95% (*p* < 0.05) and ** 99% (*p* < 0.01) confidential level.

**Table 4:** Varimax rotated component matrix for trace elements.

Variable	Components		
	Factor 1	Factor 2	Factor 3
Fe	0.945	0.074	0.152
Mn	0.920	0.276	−0.016
Ba	0.874	0.164	0.045
Pb	0.871	−0.083	0.393
Se	0.424	0.341	−0.141
Ni	0.087	0.970	−0.034
Zn	0.058	0.961	−0.094
Ag	0.079	0.838	0.387
Cu	0.385	0.837	0.122
Cd	0.151	0.091	0.934
Eigenvalues	3.630	3.506	1.247
% of Variance	36.295	35.055	12.471
Cumulative %	36.295	71.350	83.822
KMO and Bartlett’s Test			
Kaiser-Meyer-Olkin Measure of Sampling Adequacy			0.756
Bartlett’s Test of Sphericity		Approx. Chi-Square	241.074
		df	45
		Sig.	0.000

## Data Availability

Not applicable.

## Supplementary Materials

The following are available online at https://www.mdpi.com/article/10.3390/ijerph192315822/s1, Figure S1: Pretreatment processes for trace element analysis, Table S1: Recoveries and precisions for target trace elements, Table S2: Linearity of calibration and equation of target trace elements, Table S3: Summary of ABS_g_, K_p_, and RfD values for each trace elements.
