# Supplementary material for: Characteristics, Possible Origins, and Health Risk Assessment of Trace Elements in Surface Waters of the Han River Watershed, South Korea
Source: Int J Environ Res Public Health. 2022 Nov 28;19(23):15822. doi: 10.3390/ijerph192315822 (PMC9741419; doi:10.3390/ijerph192315822)
Supplement: Supplementary file 1 [file ijerph-19-15822-s001.zip › ijerph-2017638-supplementary.pdf]

Supplementary Materials

Jong Kwon Im *, Yong Chul Cho, Young Seuk Kim, Soyoung Lee, Taegu Kang and Sang Hun Kim

Han River Environment Research Center, National Institute of Environmental Research, 42, Dumulmeori-gil 68beon-gil, Yangseo-myeon, Yangpyeong-gun, Incheon 12585, Gyeonggi-do, Republic of Korea

* Correspondence: lim-jkjk@daum.net; Tel.: +82-31-770-7240

Figure and Table of Contents

Figure S1. Pretreatment processes for trace element analysis

Table S1. Recoveries and precisions for target trace elements.

Table S2. Linearity of calibration and equation of target trace elements.

Table S3. Summary of ABS_g, K_P and RfD values for each trace elements.

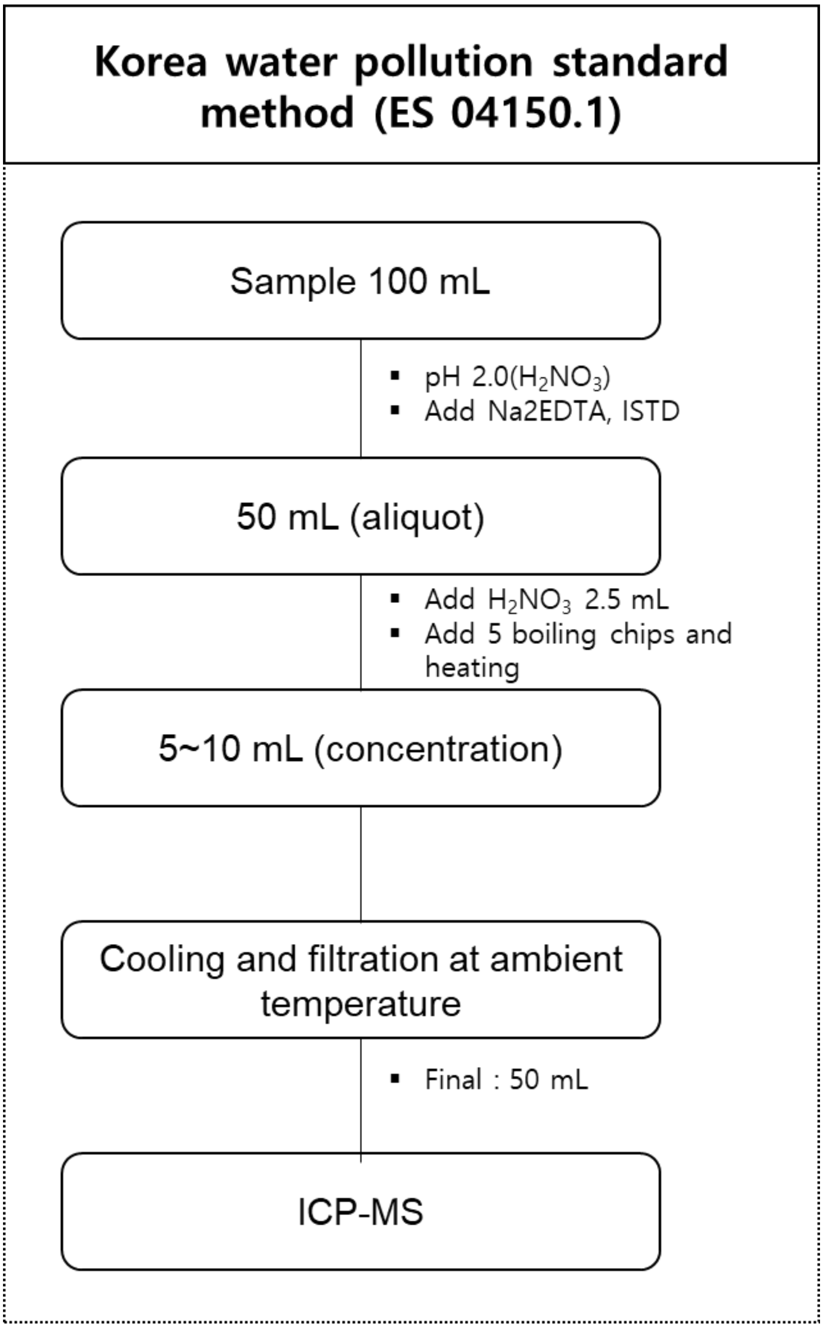

Figure S1. Pretreatment processes for trace element analysis

Table S1. Recoveries and precisions for target trace elements

Chemical Name	Spiked Conc. (μg/L)	Measured Conc. (μg/L)	Mean(μg/L) ± SD	Recovery (%)	Precision (%)
Ag	0.1	0.109, 0.110, 0.111	0.110±0.001	1.048	110.0
	1.5	1.522, 1.472, 1.482	1.492±0.026	1.775	99.4
Cd	0.1	0.102, 0.104, 0.103	0.103±0.001	1.281	103.0
	1.5	1.522, 1.465, 1.464	1.484±0.033	2.238	98.9
Cu	0.2	0.197, 0.188, 0.185	0.190±0.006	1.790	96.1
	1.5	1.310, 1.298, 1.343	1.317±0.023	1.755	87.8
Mn	0.3	0.306, 0.278, 0.283	0.289±0.015	5.150	96.3
	1.0	0.942, 0.966, 0.976	0.983±0.012	1.790	96.1
Ni	0.3	0.280, 0.281, 0.276	0.279±0.002	0.876	92.9
	1.0	0.970, 0.988, 0.993	0.983±0.012	1.217	98.3
Pb	0.1	0.090, 0.088, 0.088	0.088±0.001	1.155	88.4
	1.5	1.410, 1.373, 1.389	1.391±0.019	1.352	92.7
Zn	1.0	0.963, 0.932, 0.888	0.928±0.038	4.067	92.8
	10	9.561, 8.972, 9.142	9.226±0.305	3.305	92.4
Ba	0.1	0.099, 0.094, 0.102	0.098±0.004	4.110	98.3
	1.0	1.002, 1.012, 0.995	1.003±0.009	0.852	100.3
Fe	0.3	0.312, 0.305, 0.309	0.309±0.004	1.138	102.9
	1.5	1.512, 1.502, 1.506	1.507±0.005	0.334	100.4

Se	0.1	0.094, 0.098, 0.102	0.098±0.004	4.082	98.0
	1.0	1.005, 1.007, 0.995	1.002±0.006	0.641	100.2

Table S2. Linearity of calibration and equation of target trace elements

Compounds	Equation	Linearity(r^2)
Ag	y = 38879.0249x + 161.8629	0.9999
Cd	y = 4252.9027x + 6.7629	1.0000
Cu	y = 20666.4958x + 6120.3076	0.9997
Mn	y = 6033.0272x + 50.7390	0.9999
Ni	y = 8317.3197x + 1627.1221	0.9998
Pb	y = 72328.8696x + 1477.2238	1.0000
Zn	y = 542.9254x + 190.8678	0.9997
Ba	y = 9733.2940x - 59.6421	0.9999
Fe	y = 3393.4810x + 3592.1773	1.0000
Se	y = 113.1688x + 12.9308	0.9992

Table. S3. Summary of ABS_g, K_p and RfD values for each trace element

Heavy metal	ABS_g	K_p	RfD_{in}	RfD_d
Ag	4	$6x10^{-4}$	5	0.9
Ba	7	$1x10^{-3}$	200	14
Cd	5	$1x10^{-3}$	0.5	0.025
Cu	57	$1x10^{-3}$	40	8
Fe	1.4	$1x10^{-3}$	700	140
Mn	6	$1x10^{-3}$	24	0.96
Ni	4	$2x10^{-4}$	20	0.8
Pb	11.7	$1x10^{-4}$	1.4	0.42
Se	22	$1x10^{-3}$	5	2.75
Zn	50	$6x10^{-4}$	300	60

* ABS_g: gastrointestinal absorption factor; K_p: dermal permeability coefficient; RfD: reference dose
